# Histo-morphological and seminal evaluation of testicular parameters in diabetic rats under antiretroviral therapy: interactions with *Hypoxis hemerocallidea*

**DOI:** 10.22038/ijbms.2018.25046.6213

**Published:** 2018-12

**Authors:** Ismail Onanuga Olasile, I Ayoola Jegede, Offor Ugochukwu, O Oluwatosin Ogedengbe, Edwin CS Naidu, I Aniekan Peter, Onyemaechi O Azu

**Affiliations:** 1 Discipline of Clinical Anatomy, School of Laboratory Medicine and Medical Sciences. Nelson R Mandela School of Medicine, University of KwaZulu-Natal, South Africa; 2 Department of Anatomy, Faculty of Medicine and Pharmaceutical Sciences, Kampala International University, Tanzania; 3 Department of Anatomy, School of Medicine, The Copperbelt University, Kitwe, Zambia; 4 Department of Anatomy, School of Medicine, University of Namibia, Windhoek, Namibia

**Keywords:** Experimental diabetes, HAART, Hypoxis hemerocallidea Seminal fluid, Testis

## Abstract

**Objective(s):**

Broad range of metabolic changes associated with highly active antiretroviral therapy (HAART) has been reported over decades including reproductive perturbations. The current study aimed at investigating the role of *Hypoxis hemerocallidea (Hyp)* in the seminal and morphometric alterations in the testes of streptozotocin-nicotinamide-induced diabetic rats under HAART.

**Materials and Methods:**

Sixty-two adult male Sprague-Dawley rats were divided into A-H groups, containing 6 rats in the control group A and 8 rats in the treatment groups B-H. Diabetes was induced by intraperitoneal injection of nicotinamide (110 mg/kg BW) followed by streptozotocin (45 mg/kg BW). The animals were then subjected to various treatments with HAART, Hyp, and melatonin.

**Results:**

weights (body and testicular), histological, histochemical, seminal fluid, and morphometric analyses were carried out. Sperm count and motility were reduced in HAART (*P<*0.05/0.003) and Hyp200 (*P<*.003) groups compared with normal and diabetic controls, respectively. Sperm count was higher (*P<*.003) in HAART+ Mel and HAART+Hyp100 groups. Morphometry showed the reduction in germinal epithelium height and basement membrane thickness (*P<*.003) in the Hyp100 group compared with diabetic controls. Adjuvant use of *Hyp* and melatonin with HAART did not significantly raise these indices (*P>*.05). Histological slides showed gross distortions in HAART, diabetic and HAART +Hyp groups with marked atrophy in tubules, germ cell loss and areas of focal depletion of the cell. PAS staining revealed detached basement membrane in diabetic groups with strong PAS-stain.

**Conclusion:**

The use of *Hyp* or melatonin does not ameliorate the testicular damages in diabetic animals under antiretroviral therapy.

## Introduction

Compelling evidence from studies (1-4) indicates a consistent rise in the incidence rate of diabetic comorbidity in people living with HIV/AIDs (PLWHAs) from 5.72 to 23.8 per 1000 person-years. This is mirrored by a broad range spectrum of metabolic alterations resulting from the use of highly active antiretroviral therapy (HAART), including changes in fat redistribution and glucose homeostasis (5, 6) even in the era of intensified quality of life-related the use of HAART (7). This metabolic variance could affect the long-term forecast as a result of continuance of insulin resistance to diabetes mellitus (DM) and subsequent risk of end-organ damage that is unrelated to HIV (8, 9).

While the major culprits in the development of type 2 DM are the protease inhibitors (PIs) through inhibition of glucose translocation by means of glucose transporter-4 in the pancreatic beta cells, the thymidine analogs (NRTIs), such as zidovudine, didanosine, and stavudine, also cause insulin resistance via augmented inflammation, free fatty acid dysregulation, regional fat re-distribution, and mitochondrial dysfunction (10). This constellation of idiosyncrasies called antiretroviral-related diabetes is unwavering with the clinical picture of T2DM, rather than T1DM (11).

The negative reproductive (testicular) complications following DM have been well researched including poor sperm quality and consequent reduced fertility (12, 13). However, the associated putative mechanism(s) are still debated with rodent-models implicating altered hormonal profiles, sperm DNA damage, abnormal progression, and oxidative stress through spermatogenesis as likely pathways (14, 15). Whereas, studies (12) with human sperm specimens from diabetic males indicate that an increased mitochondrial and nuclear damage may be the culprit. Hence, it does seem plausible that hyperglycemia may result in free radical and oxidative stress damage to sperm DNA.

To date, human reproductive function (testicular integrity) continues to attract the attention of both clinical and experimental investigators as the biological need to continue procreation exists. Therefore, in resource-limited settings where access to insulin (first-line therapy for severe hyperglycemia in the presence of lipodystrophy syndrome or for patients with contraindications to oral anti-diabetic drugs) may be poor, alternative use of plant-based adjuvants to mitigate either diabetic or HAART-related side effects has been on the rise. The relative accessibility, low cost, and perceived well-being are contributory factors driving the popularity of herbal adjuvants despite uncertainties surrounding the biologically active components (16).

*Hypoxis hemerocallidea, *also known as African potato is a genus of flowering plants belonging to the family *Hypoxidaceae* with an estimated 90 species worldwide. The plant is almost cosmopolitan, growing in Southeastern Asia, Australia, Africa, and North and South America. In Africa, the genus is widespread in the south of the Sahara, with a concentration of about 41 species in southern Africa (17). The unscientific use of this plan predates many generations (18), especially, its traditional use for the treatment of a number of ailments associated with the urogenital system (19). However, the popularity of this plant soared immensely following the increased onus from the HIV/AIDS menace and its treatment as PLWHAs depends on the corm for its immune boosting potentials. The *H.* *hemerocallidea* corm is prominent for its hypoxide (antioxidant component) which is a subsidiary metabolite of *Hyp* (19) that is converted into rooperol in the large intestine (20). Pharmacokinetic research has stipulated that rooperol can be found in feces. The metabolites are also found in the urine and serum as its sulfuronides, mixed glucuronides, glycosides, and sulfates (21). Rooperol, when conjugated from its metabolites, was found to be cytotoxic to cancerous cells (19). The corm, containing glycosides is also used as food with low toxicity^17^ and has been well used for pharmaceutical and traditional purposes (18).

Great engrossment in the phytosterols use, which is another phytochemical component of *H.* *hemerocallidea*, for the purpose of enhancing the immune system and decreasing serum cholesterol, has led to more scientific inquiries surrounding these benefits (22). This is mirrored by the commercially accessible herbal medicines containing sterols and *H.* *hemerocallidea *extract enrichments with acclaimed efficacies against a number of diseases.

However, our previous study (23) indicated that the deduced importance of *H.* *hemerocallidea* extracts necessitates further scientific interrogation sequel to the emanating negative hepato-toxic indices. Therefore, as PLWHAs continue to patronize *H.* *hemerocallidea* products for certain reason(s), there is a need to unravel the potential interactions following antiretroviral therapy in diabetic comorbidity. Moreover, scientific literature interpreting testicular changes using stereological techniques in diabetic antiretroviral settings remains sparse. Therefore, this work is focused on investigating the role of aqueous extracts of *H.* *hemerocallidea* on the histomorphology and morphometric indices of the testes and semen quality of diabetic animals following HAART.

## Materials and Methods

***Drugs and chemicals***

Melatonin, Zidovudine and Nevirapine (Aspen), Lamivudine (3TC) and Nicotinamide were sourced from Pharmed Ltd., Durban, South Africa. Streptozotocin (Sigma-Aldrich, St. Louis, MO, USA) of analytical grade quality was procured from Capital Lab Supplies, Durban, South Africa. Glucose strips (Bayer Contour TS, Basel, Switzerland) were obtained from a local Pharmacy.

***Collection of plant material***

Corms of *H.* *hemerocallidea* were procured from a local market in Umbilo Road, Durban, KwaZulu-Natal, South Africa. The corms were authenticated (HH002-DBN-2014) at the Department of Life Science, Westville Campus, University of KwaZulu-Natal, Durban, South Africa.

***Extraction of the aqueous extract of hypoxis hemerocallidea***

*H.* *hemerocallidea* fresh corms were extracted following the procedure described by Ojewole *et. al. *(24). The corms were washed, chopped into a smaller portion, air dried (at room temperature, 25–28 ^°^C ) and further grounded to powdery form with a blender. The powdery corm was then immersed in hot distilled water and double extracted with 2.5 liters of hot distilled water (at 90–100 ^°^C) for 12 hr each. The soluble extracts were then concentrated to dryness with low pressure in a rotary evaporator (70±1 ^°^C). The crude aqueous extract was later freeze-dried (giving a dark brown and powdery residue). Aliquot parts of the extract residue were weighed and subsequently added to distilled water solution at room temperature used for the experiments.

***Animals***

Sixty-two adult male Sprague-Dawley rats (9-10 weeks old, 188.98±4.5 g) were utilized in this study. The rats were reared and conserved at the Biomedical Resources Unit of the University of KwaZulu-Natal. The rats were given humane care in a manner conforming to the Principle of Laboratory Animal Care of the National Medical Research Council and the Guide for the Care and Use of Laboratory Animals of the National Academy of Sciences (National Institute of Health Guide, 1985). The study protocol was approved by the University of KwaZulu Natal Animal Ethics Committee (Ethical clearance number: 056/15/Animals). All the rats were housed in well ventilated plastic cages having dimensions of (36 cm long × 24 cm wide and 15 cm high). They were maintained under standardized animal house conditions (temperature: 28–31 ^°^C; light: approximately 12 hr natural light per day) and were fed with standard rat pellets from (Meadow feeds a Division of Astral Operations Limited, Durban, South Africa) and given tap water *ad libitum*.

***Experimental design***

Diabetic animals (n=56) were distributed into seven treatment groups: B-H with eight animals per group and the control group (n=6); treatment was administered as follows:

• Group A served as negative control; 

• Group B served as diabetic control;

• Group C received the HAART cocktail (Zidovudine, Lamivudine & Nevirapine) using the human therapeutic dose equivalents (600 mg, 300 mg, and 400 mg/day, respectively), as adjusted for animal weight to obtain the corresponding therapeutic doses for the rat model (25);

• Group D received *HH* aqueous extract (100 mg/kg BW); 

• Group E received *HH* aqueous extract (200 mg/kg BW); 

• Group F received combined HAART and melatonin (5 mg/kg BW); 

• Group G combined HAART and *HH* aqueous extract (100 mg/kg BW), and

• Group H combined HAART and *HH* aqueous extract (200 mg/kg BW).

All administrations were done daily through orogastric gavage and lasted for 8 weeks.

***Induction of type 2 diabetes mellitus (T2DM)***

To generate the type 2 diabetes mellitus animal model, the diabetic groups were injected with single intraperitoneal dose of 110 mg/kg body weight nicotinamide (Sigma, Saint Louis, MO, USA) in physiological saline, and 15 mins later, they were injected with 45 mg/kg body weight streptozotocin (Sigma-Aldrich Chemical Company, Missouri, St. Louis, USA) dissolved in citrate buffer (pH 4.5) before intraperitoneal injection (26). After seven days, more than 20 mmol/L blood glucose concentration of diabetic rats was considered diabetic.

For the estimation of blood glucose, One Touch Ultra Mini Glucometer (Contour TS, Switzerland) was used. Blood obtained from the dorsal vein of the rats was used for the estimation of capillary blood glucose concentrations on days 0, 7, 14, 21, 28, 35, 42, 49, and 56 (data not shown).

***Food and fluid intake, and weight changes***

Food and fluid intake was checked daily during the experimental period (data not shown). Body weight-BW changes of the rats were recorded before the commencement of the treatment (the initial body weight), thereafter recorded weekly and shortly before sacrifice (final bodyweight). Following laparotomy, testicular weights-TW was measured by an electronic balance (Mettle Toledo; Microsep (Pty) Ltd, Greifensee, Switzerland). Testes (of each rat) were measured individually, the average value was then obtained for each animal, taken as one observation and expressed in grams (g).

***Sample collection***

After eight weeks of treatment, the rats were exposed to halothane for 3 mins in a gas anesthetic chamber. Following laparotomy, the testes were removed, weighed, and fixed in 10% neutral buffered formalin. Following proper fixation, the tissues were processed through paraffin embedding and stained with H&E and PAS techniques. 

***Epididymal sperm preparation and seminal fluid analysis***

Using anatomical scissors, the epididymides were macerated and minced in 0.8 ml of 1% trisodium citrate solution for 7–8 mins, to make up to total amount of 8 ml more solution was added and mixed further for about 1 min. The suspension was then diluted 1:1 in 10% buffered formalin. The spermatozoa were then counted with the aid of Biorad^®^Automated Cell Counter 1450101TC 20TM double-chambered counting slides loaded with 10 µl of the epididymal sperm solution. We made use of Azu’s procedure (27) for estimating the percentage of motile sperm cells. The estimation of sperm concentration and motility were carried out at room temperature (24 and 28 ^°^C). Sperm movement analysis was done using a standard hemocytometer and a light microscope (Olympus Co, Tokyo, Japan). Motility was expressed as the percentage of progressive motile spermatozoa, immotile spermatozoa, and dead spermatozoa. The process was repeated before obtaining the average reading.

***Histopathological examination of the testis***

Testicular tissues were sectioned (5 µm) using a Leica RM 2255 microtome for routine histological study of testicular microanatomy. Slide deparaffinization was carried out in xylene, rehydrated in graded ethanol, and rinsed in water. Slides were stained for 5 mins in hematoxylin, rinsed in water and counterstained in eosin. For seminiferous tubule basement membrane, glycogen, and neutral polysaccharides, the testicular sections were also stained with PAS (Periodic acid Schiff’). Slides were then slip-covered with DPX as mountant, avoiding trapping of air bubbles. With aid of a binocular microscope, sections were examined and images were acquired with the Nikon Eclipse 80i, Tokyo, Japan.

***Morphometric analyses***

To ensure an unbiased estimate of morphometric data, we adopted the following steps in obtaining our results. Seven vertical sections (from the equatorial and polar regions) were examined for each testis, using a systematic random scheme for the following morphometric parameters (28): seminiferous tubular diameter, seminiferous epithelial height/thickness, and basement membrane thickness. The vertical sections were carefully chosen by a systematic sampling method that safeguarded fair dispensation between the equatorial and Polar Regions. The diameters (D) of approximately 18 indiscriminately chosen seminiferous tubules (round or nearly round profiles) were measured for each slide, then followed with determination of a mean D from the average diameters (D1 and D2). Only when D1/D2 ≥ 0.85, D1 and D2 were taken (where 1.0=a perfect circle) to exclude longitudinal profiles that may show different degrees of asymmetrical shrinkage and damage along their length as earlier reported (28,29). The tubular height and diameter of the epithelium of the seminiferous tubule was scanned using a Leica SCN 400 (Leica Microsystems GmbH, Wetzlar, Germany) and measured across the minor and major axes using image analyzer Leica (DMLB) and Leica QWIN software, at 9100 magnification.

***Seminiferous tubule basement membrane (STBM)***

Sections stained with PAS were observed under a microscope with an image and video camera analyzer system (MiniVid DCE-1, *LW Scientific Inc*). The measurement was performed on thirty randomly selected STBM from three randomly taken sections and averaged (30).

***Testes size and gonadosomatic index (GSI)***

Testicular width and length were measured with the aid of digital Vernier calipers according to the method of Pahizkar (31). The prorate spheroid formula was used to estimate the size of the testes:

Testicle Size = width^2 ^x length x 0.523----------Equation 1

The gonadosomatic index (GSI) shows the percentage of body mass allotted to the testes; this was calculated using the body (BW) and TW as shown in equation 2 below.

GSI = (TW/BW) × 100)-------------------------Equation 2

***Testes volume***

The water displacement method commonly adopted for irregularly-shaped objects was used (32). Briefly, the testis was cautiously placed in a graded cylinder that was filled with a definite amount of water. The testis was then carefully placed into the graduated cylinder. The testicular volume (V_t_) was determined by subtracting the initial (Vi) from the final (Vf) volume of water as in equation 3 below.

V_t_=V_f_ - V_i_ (cm^3^) -----------------------------------Equation 3

***Leydig cell morphometry***

Leydig cell volume was estimated from the nuclear volume. To measure the nuclear volume, the diameters of 30 spherical nuclei with distinct nucleoli were measured for each animal (30). The nuclear volume of the Leydig cell was expressed in µm^3^ and calculated using the formula below:

Leydig_V = _(4/3)πR^3^ -------------------------------Equation 4

where R = nuclear diameter/2

***Statistical analysis***

Continuous variables were collated, analyzed, and expressed as mean+standard deviation (SD). Using one-way analysis of variance (ANOVA), statistical differences between the experimental and control groups were calculated with GraphPadInStat Software (version 6.00, GraphPad Software, San Diego, Califonia, USA), followed by Tukey-Kramer multiple comparisons test and *P*<0.05 was considered statistically significant.

## Results

***Mortality***

In the course of the experiment, a rat died in the HAART-treated group.

***Food and fluid intake and weight change***

Diabetic rats presented various symptoms often associated with diabetes (such as polydipsia, diarrhea, and polyuria). While a significantly higher fluid intake was noted, the food intake was not different in all diabetic groups in comparison with the negative control group (data not shown). The initial body weights of rats in all groups was less than the final weight with a significant lowest percentage weight change in groups G and H (*P*<0.001) compared with group A (negative control). Similarly, there was significant reduction in TW in the HAART+melatonin-treated group (*P*<0.0001) as shown in Table 1.

**Figure 1:**
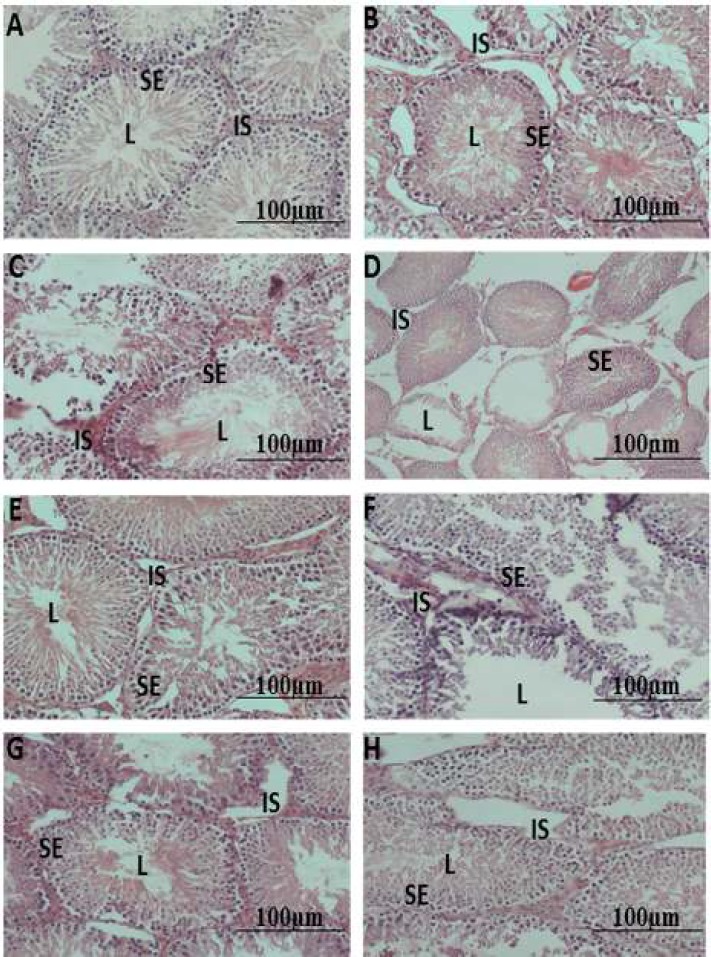
Representative photomicrographs of the seminiferous tubules in rats, (a): Negative control, (b): diabetic-DBT control, (c): DBT+ART, (d): DBT+100 mg HH, (e): DBT+200 mg HH, (f): DBT+ART +melatonin, (g): DBT+ART+100 mg HH, (h): DBT+ART+200 mg HH (H&E Staining). L=lumen, SE=seminiferous epithelium, IS=interstitial space

**Table 1:** Weight changes

**GRP**	**Treatment**	**BWi (g)**	**BWf (g)**	**BW diff(g)**	**%BW diff**	**TW (g)**	**TBWR**
**A**	Control	173.83±9.20	289.83±23.41	116.00	66.73	3.06±0.24	1.10
**B**	D-control	209.13±14.17	270.63±60.76	61.50	29.41	3.03±0.26	1.10
**C**	HAART-d	213.25±14.25	268.25±40.91	55.00	25.79	3.17±0.37	1.10
**D**	Hyp_100_	196.25±18.61	280.50±53.70	84.25	42.93	3.19±0.23	1.10
**E**	Hyp_200_	187.63±20.68	242.38±50.20	54.75	29.18	3.26±0.43	1.13
**F**	HAART+Mel	181.13±19.75	231.25±59.05	50.12	27.67	1.96±1.24^αβ^	0.80^αβ^
**G**	HAART+Hyp_100_	186.88±56.42	214.25±26.44	27.37	14.65^α^	3.04±0.36	1.14
**H**	HAART+Hyp_200_	186.75±17.47	213.00±52.10	26.25	14.06^α^	2.84±0.46	1.13

Data are presented as the mean ± SD; *P*<0.0001^αβ ^vs Group A^α^ and *P*<0.05 Group B^β^ (Tukey-Kramer multiple range *Post-hoc* test). BWi = body weight-initial, BWf = body weight-final, BW = Body Weight, TW = Testicular Weight.

**Table 2:** Seminal fluid analysis

**GRP**	**Treatment**	**Sperm count (x10** ^6^ **)**	**Motility (%)**		
			**Motile**	**Immotile**	**Dead**
**A**	Control	46.78±3.07	64.33±7.42	19.33±7.45	16.33±5.85
**B**	D-control	10.83±2.00^α^	44.50±4.98	25.754.71	28.75±4.24
**C**	HAART-d	12.86±3.32^α^	22.00±8.55^αβ^	25.00±8.21	55.25±5.17^αβ^
**D**	Hyp_100_	27.43±3.94	28.25±9.82^α^	22.75±9.68	46.50±5.84^α^
**E**	Hyp_200_	25.90±4.54	16.00±3.80^αβ^	23.75±8.84	60.25±5.90^αβ^
**F**	HAART + Mel	32.60±8.48^β^	48.40±5.90	9.20±5.76^β^	43.60±9.26^α^
**G**	HAART + Hyp_100_	34.45±5.66^β^	55.50±3.56	20.00±7.33	23.25±2.10
**H**	HAART + Hyp_200_	21.63±1.71^α^	43.50±3.87	30.25±6.21	25.00±4.16

Data are presented as the mean ± SD; *P*<0.0001 vs Group A^α^ and *P*<0.003 Group B^β^ (Tukey-Kramer multiple range *Post-hoc* test).

**Table 3:** Testicular morphometric indices in experimental groups

**GRP**	**Treatment**	**TL (mm)**	**TWd (mm)**	**GSI (%)**	**STD (µm)**	**SEH (µm)**	**STBM (µm)**
**A**	Control	19.90±0.9	12.12±0.4	1.06±0.1	293.5±2.7	93.25±5.6	1.74±0.01
**B**	D-control	20.21±0.8	12.03±0.7	1.15±0.2	259.0±4.3^α^	78.13±3.7^α^	2.42±0.07^α^
**C**	HAART-d	20.41±0.8	12.36±0.8	1.19±0.2	263.6±3.1^α^	76.63±2.6^α^	2.49±0.21^α^
**D**	Hyp_100_	20.04±0.9	11.43±0.4	1.17±0.2	267.5±4.7^α^	66.25±3.7^αβ^	1.95±0.02^β^
**E**	Hyp_200_	20.98±1.1	12.45±1.0	1.37±0.2	265.1±3.6^α^	76.75±2.9^α^	1.94±0.04^ β^
**F**	HAART+ Mel	17.16±3.2^αβ^	9.68±2.5^αβ^	0.77±0.4	271.4±3.7^α^	84.00±2.5^α^	2.58±0.20^α^
**G**	HAART+ Hyp_100_	20.50±1.0	12.03±0.7	1.42±0.1^α^	241.8±2.7^α^	78.25±1.9^α^	2.48±0.17^α^
**H**	HAART+ Hyp_200_	19.85±1.1	11.76±0.8	1.36±0.2	242.3±3.9^α^	75.75±2.6^α^	2.53±0.20^α^

Data are presented as the mean ± SD; *P*<0.001 vs Group A^α ^and Group B^β^ (Tukey-Kramer multiple range *Post-hoc* test), GRP = group, TL= testis length, TWd = testis width, GSI = gonadosomatic index, STD = seminiferous tubule diameter, STBM=seminiferous tubule basement membrane, SHE = seminiferous epithelium height.

**Table 4:** Testicular size, volume, and Leydig cell morphometry

**GROUP**	**Treatment**	**TS (mm)**	**TV (mm** ^3^ **)**	**LCND (µm)**	**LCNV (µm** ^3^ **)**
**A**	Control	1532±62.17	3.21±0.31	24.63±1.51	1314±85.41
**B**	D-control	1529±48.77	3.24±0.15	20.50±2.39	893±77.45
**C**	HAART-d	1640±79.31	3.26±0.32	19.50±2.83	683±54.50
**D**	Hyp_100_	1369±38.61	3.53±0.23^β^	24.75±2.55	1359±74.90
**E**	Hyp_200_	1717±61.70	3.50±0.28^β^	24.00±2.07	1229±58.30
**F**	HAART + Mel	1453±99.70^β^	3.19±0.65	28.13±2.10	1967±73.70
**G**	HAART + Hyp_100_	1562±86.11	3.47±0.34	23.25±4.92	1220±23.30
**H**	HAART + Hyp_200_	1450±95.80	3.38±0.30	19.88±3.18	732±66.70

Data are presented as the mean ± SD; *P*<0.0001 vs Group B^β^ (Tukey-Kramer multiple range *Post-hoc* test), TS = testicular size, TV = testicular volume, LCND = Leydig-cell-nuclear-diameters, LCNV = Leydig-cell-nuclear-volume.

**Figure 2:**
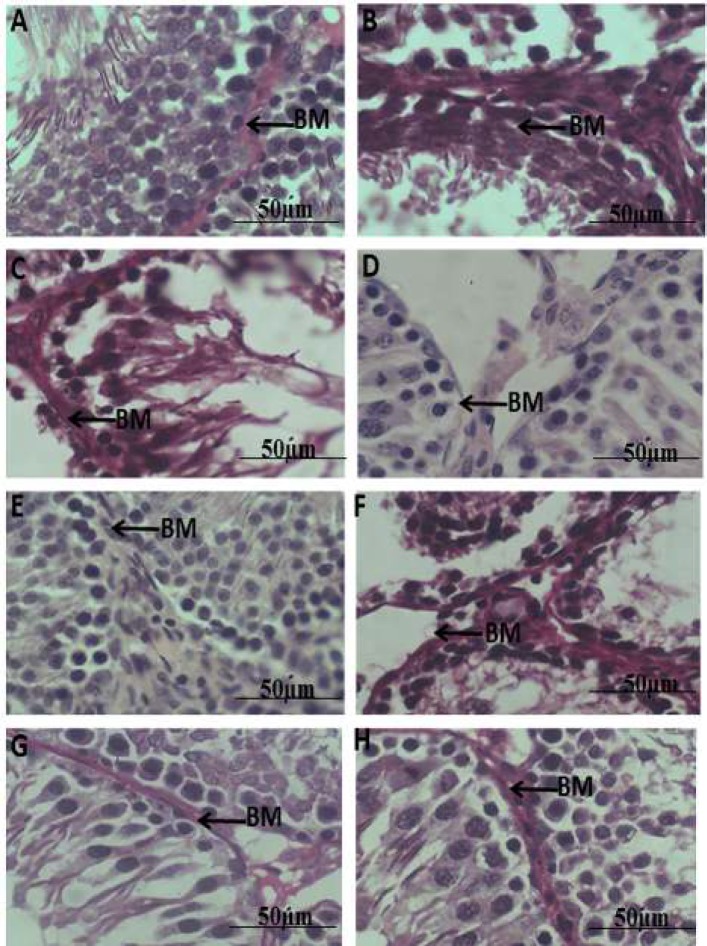
Representative photomicrographs of the seminiferous tubules in rats, **(a)**: Negative control, **(b)**: diabetic-DBT control, **(c)**: DBT + ART, **(d)**: DBT + 100 mg *HH, ***(e)**: DBT + 200 mg *HH, ***(f)**: DBT + ART + melatonin, **(g)**: DBT +ART +100 mg *HH, ***(h)**: DBT + ART + 200 mg (PAS Staining). BM = basement membrane.

***Seminal fluid analysis***

Sperm counts in diabetic (positive control) and HAART-alone groups were significantly lower than the negative control with proportionate declines in motility as well (*P*<0.0001). Though the sperm count for groups treated with *H.* *hemerocallidea* at both doses was higher than diabetic controls, these values were not significant (*P*>0.05). Sperm motility and number of dead sperms were reduced (*P*<0.003) in the higher dose of *H.* *hemerocallidea*. However, sperm count in groups F and G receiving adjuvant melatonin and Hyp_100_ with HAART were significantly higher (*P*<0.003) in comparison with the positive control, and sperm motility was essentially higher but not significantly different from both negative and positive controls (Table 2).

***Morphometric indices***

Testicular gonadosomatic index (GSI) was essentially not significantly different in any group from either control except in groups F and G (lower but not significant) and significantly higher (*P*<0.0001), respectively. Groups treated with both doses of *H.* *hemerocallidea* recorded significant reduction (*P*<0.0001) in basement membrane thickness of seminiferous tubules with the lower dose group also recording equivalent decrease in epithelial height (when compared with positive controls). The seminiferous tubule diameter and epithelial height in groups B, C, and D rats all presented with various levels of decline when compared to the negative controls (group A) (*P*<0.001). However, the values of the basement thickness were higher in groups B and C (*P*<0.001) but lower in group D (*P*<0.001) compared with group A (positive controls). In groups G and H (adjuvant treatment with both doses of *H.* *hemerocallidea* and HAART) these parameters were not significantly different from values of the positive control group (*P*>0.05) (Table 3).

***Leydig cell morphometry***

Testicular volume was significantly elevated in groups D and E compared with positive controls (*P*<0.001). There were no changes in the average Leydig cell nuclear volumes nor in the number of these cells in the testis in any group except in the diabetic control group (with lower LCNV from negative controls) but this was insignificant (Table 4).

***Histological evaluation of the testes***

H&E sections of negative control testes showed normal, closely related seminiferous tubules with basement membranes intact and clearly delineated. The spermatogenic series was complete with all the spermatogenic cells in layers and lumen populated with immotile sperm cells. There were no obvious infiltrations in interstitial spaces (Figure 1A). Diabetic control (positive) testis section displayed highly irregular basement membranes in many seminiferous tubules with disruptions in spermatogenic series and atrophic changes seen in some tubules. The lumen of many tubules showed the presence of cellular debris (Figure 1B). Many tubules in the HAART group were necrosed with detached/denuded spermatogenic cells from the basal lamina (Figure 1C). In testicular sections of groups D and E, numerous tubules were extensively atrophied and just lining the remaining basement membrane, with interstitial spaces sparsely populated with cellular components (Figure 1D). These changes were seen less in group E, which showed better histologic preservation and outlines (Figure 1E). Histologic sections from testis in groups treated with adjuvant melatonin and both doses of *H.* *hemerocallidea* displayed seminiferous tubules with better architectural preservation in groups G, H, and F (in that order). In many tubules, there was evidence of loss of germinal cells series most marked in group F but the interstitial spaces appeared normal (Figures 1F, G, and H).

PAS staining of the testicular cross-section in normal controls (negative control A) showed seminiferous tubules with the regular course of spermatogenesis and a basement membrane that is delineated with positive PAS stain (pink). The nuclei of the spermatogenic cells are stained dark and appear normal in layers, and interstitial spaces were normal also (Figure 2A). In Figure 2B, there is a strong intense positive PAS reaction seen in the basement membranes of tubules (and capsule not shown) with many tubules showing focal detachment of germ cells from lamina as well as reduced epithelial height. Changes seen in Figure 2C included atrophy with loss of germ cells. Basement membrane was positive for PAS and also a strong PAS reaction of debris in the tubular lumen was present. In testicular sections from Figures 2 D&E, the basal lamina was not strongly stained (poor PAS reaction) but the spermatogonia were visible and identified by their densely stained nuclei (2D) whereas, in 2E, the tubular cellular contents and interstitium were normal. A strong PAS reaction was seen in basement membrane lining of the testis of group F with denudation of sperm cells from the basement membrane (Figure 2F). Figures 2 G and H equally showed a clear basement membrane and cellular content in tubules but these were better preserved in H rather than G.

While seminiferous tubules basement membrane (STBM) in the controls (Figure 2A) was noticed to be normal, there were increments in the basement membrane thickness in the non-treated diabetic groups (Figure 2 B, C, F, G, and H). On the other hand, the thickening of the STBM in the *H.* *hemerocallidea*-treated groups (100 mg and 200 mg) were found to be less in other groups (Figures 2 D, E) as shown in Table 3. However, the thickness of the STBM was significantly reduced in the *H.* *hemerocallidea-*treated diabetic animals (100 mg and 200 mg) when compared with the diabetic animals (*P*<0.0001) (Table 3).

## Discussion

The exponential growth in the use of plant-based adjuvants in developing and developed countries of the world has continued despite in many cases unproven scientific validation of some of the perceived positive effects (23). This has posed a major concern with regards to the safety of the chemical constituents of the products and possible interactions with other compounds. The World Health Organization has listed nearly 21,000 plants used for medicinal purposes around the world including India (33), Nigeria, and South Africa (23). Some of the plants have been used for the treatment of many non-communicable diseases including diabetes mellitus as well as infectious diseases like HIV/AIDs. 

Results from this study revealed that diabetes and HAART resulted in extensive histological alterations in the seminiferous tubules of the rats; these changes were not mitigated with the treatment of adjuvant *Hyp*. The decline in the spermatogenic series was seen with atrophied seminiferous tubules and bereaved sperm cells as a result of diabetes-associated pathology (34). Further supporting this hypothesis are morphometric data indicating a significant reduction in the seminiferous epithelial height as well as increased thickness of the basal lamina in diabetic animals in comparison with the normal control animals. Diabetes and HIV/AIDS have been a major crippling disease in the world with the devastating loss of lives exacerbated by worsening economic crises since the 1930s. Therefore, the reliance on plant-based adjuvants by people within Sub-Saharan Africa remains rife and may just be the succor the average person saddled with these ailments may need.

Although the blood glucose levels are not reported in the current study, in previous studies (35) varying the dosage of the methanolic extract of *H.* *hemerocallidea* was found to lower the blood glucose concentrations of normal and hyperglycemic animals by 35% and 55%, respectively when compared with anti-diabetic effects of glibenclamide. The physiological response associated with diabetes (such as weight loss, excessive loss of fluid) were observed in our current study, which adjuvant *Hyp *was able to restore. This perhaps supports the advantageous use of *H.* *hemerocallidea* extracts by the indigenous communities for the amelioration of the adult induced-diabetes mellitus (35).

Interestingly, our results on seminal fluid changes (count and motility) do indicate that the use of *Hyp *extracts (in the diabetic state) may not necessarily be helpful in mitigating the ravages of diabetes per se on these parameters (Table 2) but may likely amplify the negative effects. However, we also observed a slightly opposite effect in adjuvant *Hyp *+ HAART where the significantly reduced motility percentage in diabetic rats treated with *Hyp *was reversed (as seen by increased motility as well as a significantly elevated sperm count), especially with the higher dose of *Hyp*, compared with diabetic controls and HAART alone groups, respectively. The possible reason(s) for these aberrations are unclear; however, this may result from the direct effects of certain components in *Hyp *on these spermatogenic indices, especially in a diabetic state. Rooperol is an active component of the *Hyp *plant used in this protocol, and studies indicate that the free radical scavenging properties are ten times more than ascorbate ions (36). As spermatozoal motility is a key factor in determining the ability of any sperm cell to fertilize and this is acquired at the epididymis, the likelihood of damage to flagella structures or energy supply due to negative interactions with *Hyp *components is probable. Moreover, the pharmacological dynamics of antioxidant-prooxidant equilibrium could have tilted the balance in favor of a pro-oxidant effect of *Hyp *in this protocol. 

The disturbances in the spermatogenesis process presented in diabetic states have previously been documented (37, 38). This is also applicable to the negative testicular perturbations of HAART (39). DM causes a decrease in tubular diameter and seminiferous tubule atrophy (34) possibly linked to cell apoptosis. In the current study, most morphometric observations, especially in the diabetic+*Hyp* group (low dose), were significantly reduced (epithelial thickness 66.25±3.69 µm and tubular basement membrane thickness 1.95±0.02 µm compared with diabetic and normal controls). Similarly, the epithelial thickness (78.25±1.98 µm) and basement membrane thickness (2.48±0.17 µm) were not significantly different from the HAART diabetic group. These changes are indicative of morphologic disorders in spermatogenesis and are consistent with reports (40) that showed atrophy and reduction of seminiferous tubular diameter, with loss of Sertoli and germinal cells in diabetic animals. In addition, alterations of the basement membrane thickness resulting from diabetes are well documented, which ultimately lead to the reduction in spermatozoa production and the morphology of their tubules (41). Plausible clarification for this can be understood by the inadequacy of insulin production, where the functionality of the Sertoli and Leydig cells becomes deteriorated with weakening of spermatogenesis as a result of declined FSH levels (42).

Though the diabetic-associated pathophysiology is not entirely known, many studies have indicated the role of free radicals in its impediment and pathogenesis (43)*.* Free radicals are dexterously detrimental to the proteins, lipids, DNA, and cellular molecules resulting in an alternation of cellular function. Furthermore, hyperglycemia (as seen in STZ-induced diabetes) causes oxidative stress because of the increased level of reducing sugars that can easily react with lipids and proteins. The consequence is the increased production of oxygen reactive species that gradually leads to the development of diabetic complications (44). While many plant products have shown to have remarkable antioxidant activities that are beneficial in diabetic conditions (45), it is highly likely that *Hyp *extract components are unable to scavenge these radical species from the tissues in this study. This poses a serious concern with regards to the concomitant use of this plant extract by patients who are undergoing antiretroviral therapy in AIDS-related therapeutic programmes such as HAART (who may be diabetic as well). While the use of *Hyp *extract is widespread among these patients (46), there are obvious challenges regarding metabolism and increased possibility for consequential clinical adverse reactions, amid a reduction in efficacy for the HAART protocol per se. The metabolism of a large proportion of medicines (as used in HAART) is mediated through CYP P450 enzymes in the liver, particularly, CYP3A4. This has broad substrate specificity. Notable inhibition of CYP3A4 can have a detrimental effect on the safety of *Hyp* and could potentially interact with CYP3A5 enzymes, incidence of which is typically higher amidst this region’s populace (47).

Qualitative data on light microscopic examination (H&E and PAS stains) of sections also indicate that testicular sections of the diabetic group, as well as HAART-alone group, were essentially deranged. Mirroring this closely was the exacerbation seen in diabetic *Hyp *groups as well, with atrophied tubules and loss of cellular components. It is important to stress the significance of cellular junctions in the maintenance of the integrity of the testicular tissue. Therefore, diminution in tubular size is closely linked with detachment and loss of germ cells as gleaned from both the H&E and PAS-stained sections of diabetic groups as well as *Hyp *groups. Administration of adjuvant *Hyp *or melatonin with HAART could not fully mitigate these deficits. Reduction in Sertoli cells causes reduction in sperm numbers due to their role in spermatogenesis, providing physical and nutrition protection and necessary hormone signals for successful spermatogenesis (48). Therefore, when Sertoli cell numbers reduce, the number of germinal cells decreases intensively (49) as seen in many of the tubules described in this report.

Treatment with *Hyp *does not alter the morphometry of Leydig cells except an observational increase in the Leydig cell nuclear volume of diabetic animals treated with HAART concomitantly with melatonin (30, 50). This was attributed to drug-drug (HAART-melatonin) interactions, which could probably suppress the efficacy on cell proliferation and differentiation properties of melatonin. This still remains to be fully investigated in subsequent phases of this work. 

The rapid elevation in the blood melatonin concentration following administration of melatonin resulting from its hydrophilic and lipophilic properties (34) enabled its rapid passage through all biological membranes into the cellular as well as and their subcellular constituents. This is advantageous for melatonin over certain antioxidants that penetrate cells at a slow pace. However, the administration of melatonin (5 mg/kg) in this protocol did not ameliorate the resulting testicular dysfunction. This presumably may result from melatonin-HAART interaction, suppressing its efficacy as a potent antioxidant.

## Conclusion

The present study shows that *Hyp *or melatonin use does not mitigate the testicular damage in diabetic male rats in the era of antiretroviral therapy. To our understanding, this is the first report suggestive of the inability of *Hyp *to mitigate testicular distortions in a diabetic model of HAART and the potential for negative drug-drug interactions. Therefore, adjuvant use of *Hyp *by PLWHAs should be cautious in view of these observations till further details are known.
